# Review of Methods for Detection *Helicobacter pylori* in Kazakhstan

**DOI:** 10.1002/jgh3.70101

**Published:** 2025-01-17

**Authors:** Lavrinenko Alyona, Seisenbekova Aizhan, Turemuratova Aidana, Shkreba Alexey, Yukhnevich Yekaterina

**Affiliations:** ^1^ Laboratory of the Institute of Life Sciences NJSC Karaganda Medical University Karaganda Kazakhstan; ^2^ Department of Internal Diseases Karaganda Medical University Karaganda Kazakhstan; ^3^ Scientific Research Laboratory of the Institute of Life Sciences NJSC Karaganda Medical University Karaganda Kazakhstan; ^4^ University Clinic NJSC, Karaganda Medical University Karaganda Kazakhstan; ^5^ Karaganda Medical University Karaganda Kazakhstan

**Keywords:** *Helicobacter pylori*, Kazakhstan, microbiological cultivation, molecular biological methods, serological tests

## Abstract

*Helicobacter pylori* (*H. pylori*) infection can cause a wide range of gastrointestinal disorders, including chronic nonatrophic gastritis, multifocal atrophic gastritis, peptic ulcer disease, gastric adenocarcinoma, and extra‐nodal B‐cell lymphoma. Although the prevalence of *H. pylori* infection has decreased among adults, it is still very common. Approximately 90% of gastric adenocarcinomas are associated with *H. pylori* infection. Despite the established link between *H. pylori* infection and noncardiac gastric cancer, and the increasing incidence of gastric cancer in Kazakhstan, there are limited data on the prevalence of *H. pylori* in the country. This may be due to the difficulty of detecting *H. pylori* and the unavailability of diagnostic methods. This review presents the current data of diagnostic tests for the detection of *H. pylori* in Kazakhstan with a focus on limitations and practical significance.

## 1 Introduction

The infection of *Helicobacter pylori* (*H. pylori*) causes chronic gastritis through Correa's cascade and can improve to severe gastroduodenal pathologies, including peptic ulcer disease, gastric adenocarcinoma, and lymphoma of lymphoid tissue associated with the gastric mucosa [1, 2, 3]. This pattern of progression highlights the importance of early detection and treatment of *H. pylori* infection, especially in individuals who have a history of infection [4, 5]. However, *H. pylori* is often asymptomatic [6]. Early detection and treatment can help prevent disease progression and reduce the risk of complications [7].

The prevalence of *H. pylori* infection varies depending on factors such as age, ethnic background, comorbidities, living region, socioeconomic status, and hygienic conditions [8, 9, 10]. Although the incidence of *H. pylori* infection in adults is decreasing [11], the global prevalence of *H. pylori* infection is still approximately 43.1%, according to a meta‐analysis and systematic review that analyzed data from 2011 to 2022 stratified by the World Health Organization (WHO) regions [12].

*H. pylori* has several virulence factors that can contribute to the development of stomach cancer [13, 14, 15]. According to the latest data, Kazakhstan is one of the top 10 countries with the highest incidence of gastric cancer [16, 17]. Over the past decade (2009–2018), 19 672 people in Kazakhstan died from gastric cancer. The average annual mortality rate was 13.2 per 100 000 people, and the 5‐year survival rate for patients with gastric cancer was less than 20% [18, 19].

Despite the well‐established link between *H. pylori* infection and gastric cancer, as well as the increasing burden of gastric cancer in Kazakhstan, there is still a lack of data on the prevalence of *H. pylori* in the country. In Kazakhstan, clinical guidelines follow the international Maastricht guideline, which calls for standardized treatment. This negative trend may be due to difficulties in diagnosing, detecting *H. pylori*.

In this regard, the aim of the study is to analyze the most commonly used methods for detecting/diagnosing *H. pylori* infection in Kazakhstan.

## 2 Search Method

The Web of Science, Google Scholar, and PubMed databases were used to systematically search for articles. As it was expected that there would be limited data available, we included articles with no time limits or specific types of research. Case reports and case series were excluded. Local journals were also searched for published studies on methods of detecting *H. pylori* infection in Kazakhstan. Papers were required to evaluate the frequency of positive and negative results to be eligible for inclusion in this review. Cross‐sectional studies were also considered.

Literature published for all time was systematically identified in the database using the following search terms: ( *Helicobacter pylori* ) AND (detection) AND (serological tests OR microbiological cultivation OR urease breath test OR histology OR rapid urease test OR stool antigen test).

## 3 Methods for Detecting *H. pylori*

Generally, methods for detecting *H. pylori* can be classified into two categories: invasive and noninvasive [20, 21, 22]. Noninvasive tests include breath tests, serological and stool antigen tests, while endoscopy with a biopsy is the main invasive method. The choice of a specific test depends on several factors, including availability of the test, equipment in the laboratory, and clinical condition of the patient.

For the Republic of Kazakhstan, the articles did not provide information on the criteria for *H. pylori* diagnostic tests (sensitivity, specificity, accuracy, positive predictive value, and negative predictive value). Therefore, we relied on global data for these tests. Additional comparative characteristics of different *H. pylori* diagnostic methods are summarized in Table 1.

**TABLE 1:** World review of *H. pylori* diagnostic methods.

Test		Sensitivity (%)	Specificity (%)	Accuracy (%)	PPV (%)	NPV (%)	Reference
UBT	Gab Control *H. pylori* (gabmed GmbH, Cologne, Germany)	91.4	76.7		65.3	94.9	[23]
	E‐plate Eiken *H. pylori* AntibodyII (Eiken Chemical Co. Ltd., Tokyo, Japan)	97.4	76.3	91.6	91.5	91.8	[24]
	Cer Test *H. pylori* blister test (Cer Test Biotec S.L., Zaragoza, Spain)	68.7	97.6	91.5	88.5	92.0	[25]
SAT	Via Sure *H. pylori* real‐time PCR detection kit (Cer Test Biotec S.L., Zaragoza, Spain)	85.7	100	92	100	84.6	[26]
	URINELISA (Otsuka Pharmaceuticals Co. Ltd., Tokyo, Japan)	85.5	88.8				[27]
RUT	*H. pylori* Ig GEIA test kit (Rapid Labs Ltd., Little Bentley, Essex, UK)	90.5	39.6	61	52.1	85.2	[28]
	Quick Chaser *H. pylori*, QCP (Misuho Medy, Tosu, Japan)	92.3	100				[29]
Culture test	Amplified IDEIA Hp StAR (Thermo Fisher Scientific, Waltham, MA, USA)	93.6	100	96	100	87.3	[30]
	Rapirun (Otsuka Pharmaceutical Co. Ltd., Tokyo, Japan)	86.2	90.8	89.4	80.6	93.7	[31]
Histology	Recom Line *H. pylori* IgG (Mikrogen Diagnostik, Germany)	96.1	20.9		69.7	73.7	[32]
	Gastro Panel (Biohit Oyj, Helsinki, Finland)	74.7	95.6		91	86	[33, 34]
ELISA	Commercial ELISA (AccuBind ELISA, Monobind, USA)	96.7	42.8	82.9	83.1	81.8	[35]

Abbreviations: ELISA, enzyme‐linked immunosorbent assay; NPV, negative predictive value; PPV, Positive predictive value; RUT, rapid urease test; SAT, stool antigen test; UBT, urease breath test.

Also, all of these techniques have their own advantages and limitations for Kazakhstan. These data are presented in Table 2.

**TABLE 2:** Comparative characteristics of diagnostic methods of *H. pylori*.

No.	Diagnostic tests	Advantages	Disadvantages
1	UBT	Availability in Kazakhstan, average cost 16$, the possibility of taking the test in private laboratories and large medical clinics and centers in Kazakhstan, popularity of the test noninvasiveness, high specificity, and sensitivity.	Lack of opportunity to get tested in the village, the possibility of false‐negative results (taking PPIs, antibacterial drugs, bismuth drugs, gastrectomy, the presence of extensive atrophy or metaplasia of the gastric mucosa in the antrum), more expensive than determination of *H. pylori* antigen in stool.
2	SAT	Availability in Kazakhstan, average cost 10$, the possibility of taking the test in private laboratories and large medical clinics and centers in Kazakhstan, popularity of the test, noninvasiveness, accessibility, convenience of sample collection, the ability to store the sample at freezing temperature, does not require expensive chemicals and special equipment, the price is lower than other noninvasive methods, does not require fasting, when using new monoclonal antibodies, does not require PPIs withdrawal.	Lack of opportunity to get tested in the village, the possibility of false‐negative results (excretion of antigen may vary over time, antigen may be destroyed during passage through the intestines), low accuracy compared to UBT (use of a mucolytic agent like N‐acetylcysteine, bismuth‐based drugs, low bacterial colonization in the stomach, and gastrointestinal bleeding).
3	ELISA	Availability in Kazakhstan, average cost 10$, the possibility of taking the test at clinics, the cost is covered by the state, popularity of the test, convenience of sample collection, are less traumatic than PCR.	The possibility of false‐positive results (cross‐reacting antigens) does not allow for distinguishing an active infection from a previous *H. pylori* infection.
4	RUT	Availability in Kazakhstan, average cost 15$, included in the guaranteed amount of free medical treatment, the cost is cheaper taking into account the endoscopy procedure than UBT with ELISA. In the world, on the contrary, UBT is widely available and cheaper.	Included in the list of guaranteed volume of medical care along with endoscopy, but due to its invasiveness, it is not very popular, the possibility of iatrogenic infection of patients with *H. pylori* , the possibility of false positive results (the presence of other urease‐producing bacteria in the stomach), the possibility of false‐negative results (atrophy of the gastric mucosa, recent use of antibacterial drugs, PPIs, acute phase of gastric ulcer).
5	Culture‐based test	High specificity, ability to determine sensitivity to antimicrobial medicine.	Not available in Kazakhstan, only in grant‐funded studies, invasiveness, the possibility of infection of patients with *H. pylori* iatrogenically, the duration of obtaining results (2–3 weeks), the possibility of false‐negative results (low bacterial load, use of PPIs, antibiotics, alcohol, presence of bleeding, violation or inaccurate adherence to culture techniques, poor quality of samples, delay in transportation, exposure to aerobic environment, and lack of microbiologist qualifications).
6	PCR in biopsy material on *Н. pylori* DNA	Ability to determine sensitivity to antimicrobial medicine, high sensitivity, and specificity, ability to identify *H. pylori* in small samples, does not require any special materials for processing or transportation, rapid results, ability to identify various genotypes of bacteria.	Not available in Kazakhstan, only in grant‐funded studies, invasiveness, the possibility of iatrogenic infection of patients with *H. pylori* due to insufficient disinfection of endoscopes; the possibility of false‐negative results (biopsy specimens that do not contain *H. pylori* may be included in the study).

Abbreviations: DNA, deoxyribonucleic acid; PPI, proton pump inhibitor.

In total, seven studies on the diagnosis of *H. pylori* were detected over the entire search period in Kazakhstan. These studies are summarized in Table 3. It should be noted that some of these studies had limitations, with the main limitation being the small size of the study population, which reduced the statistical power of the studies.

**TABLE 3:** Local studies of *H. pylori* infection in Kazakhstan.

No.	Authors of the study	Diagnostic test	Number of participants (*n*)	Region	Positive participants (*n*)	Positive participants (%)	Reference
1	Nurgalieva Z., co‐authors, 2002	ELISA	103	Southern Kazakhstan	76	74.3	[21]
2	Zhangabylov А., co‐authors, 2002	ELISA	288	Southern Kazakhstan	230	80	[36]
3	Aldiyarova M., 2011	UBT	446	Southern Kazakhstan	254	57.6	[37]
4	Kulmagambetova G., co‐authors, 2011	PCR, microbiological cultivation	19	Central Kazakhstan	16	84.2	[38]
5	Abylkasymova К., 2012	PCR	52	Southern Kazakhstan	43	91.7	[39]
6	Nasipov S., Kuanov E., 2015	ELISA	520	Western Kazakhstan	520	100	[40]
7	Mežmale L., co‐authors, 2021	ELISA	166	Western Kazakhstan	104	62.7	[41]

The invasion of *H. pylori* triggers a widespread immune reaction. Antibodies against *H. pylori* emerge in the bloodstream approximately 3–4 weeks after infection. These antibodies can be identified using one of three techniques: enzyme‐linked immunosorbent assay (ELISA), latex agglutination tests, and Western blotting [21]. According to an analysis of published data on *H. pylori* diagnosis, the ELISA is the most commonly used method in Kazakhstan. In 57.3% of the study cases, serological testing was used from 2001 to 2021. According to these studies, the overall prevalence of *H. pylori* infection was 74.3%, 80%, and 62.7% in 2002 and 2021, respectively [36, 37, 38, 39, 40, 41, 42]. *H. pylori* screening methods, such as ELISA, allow multiple individuals to be tested at the same time. In addition, ELISA is currently the most inexpensive and accessible method in Kazakhstan, with an average cost of $7.29 in laboratories. Furthermore, patients with suspected *H. pylori* infection, within the framework of guaranteed medical care, can undergo this test free in clinics.

Serological tests based on the detection of antibodies to *H. pylori* are often used to screen for *H. pylori* infection due to their rapid results and cost‐effectiveness [28]. These tests are used for epidemiological purposes because they provide rapid results, but they cannot distinguish active infection from previous infections because antibodies to *H. pylori* persist for a long time.

The specificity and sensitivity of serological testing can differ. One meta‐analysis revealed that the specificity and sensitivity of the test were 85% and 79%, respectively. Another investigation revealed that the accuracy ranged from 76% to 84%, while the precision ranged from 79% to 90% [43]. In general, there are a lot of studies with conflicting results.

In general, unifying criteria for serological tests depend on the quality of the antigen used [44], and since *H. pylori* strains may vary regionally, local validation of the test is necessary [45].

The urease breath test (UBT) is useful for both initial diagnosis and detection of active *H. pylori* infection, as well as post‐treatment assessment [46].

Its advantages include high sensitivity and specificity, with sensitivity above 97% and specificity above 93%. The accuracy is also above 91%, the positive predictive value above 85%, and the negative predictive value is over 91% [24, 47]. The test is noninvasive, but there are some disadvantages. First, there is a likelihood of a false‐negative result, which is not recommended for patients taking proton pump inhibitors (PPIs), antibiotics, or those with bleeding ulcers or undergoing gastric surgery [24, 48]. Additionally, widespread atrophic gastritis can also lead to false‐negative results. Other limitations include the need for special equipment to measure carbon dioxide and handling of radioactive materials, which results in a higher cost for the test [49, 50]. UBT also is the most commonly used method for *H. pylori* diagnosis in Kazakhstan. Due to its noninvasive nature and fast performance, this method has become widely available and popular in cities throughout Kazakhstan. Although it costs more than the ELISA test, averaging $16 per test, it remains popular owing to its accuracy and ease of use. In 2011, M. Aldiyarov conducted a larger study in southern Kazakhstan, finding *H. pylori* in 57.6% of participants using a breath test. A total of 446 people from Southern Kazakhstan participated in this study [37]. The practical significance of the breath test is not only its ability to diagnose *H. pylori* , but also its use to evaluate the effectiveness of eradication therapy.

Polymerase chain reaction (PCR) is a highly sensitive and intelligent method [51] that can detect *H. pylori* in small quantities in samples [52]. It also allows different genetic species to exist [53]. However, one limitation of PCR is that it can mistakenly detect dead DNA fragments in the stomach lining of patients after treatment, leading to possible false‐positive results [54]. In two of the trials, *H. pylori* was identified using DNA determination in a biopsy sample of the stomach lining using the PCR method. Although endoscopy for upper gastrointestinal diseases is generally available in Kazakhstan at the primary healthcare level, the PCR method for *H. pylori* detection is only used in scientific studies owing to technical limitations. This explains the small number of studies that have used this method. Despite the limited number of studies, this research method remains relevant in Kazakhstan as it is necessary to sequence samples after PCR to determine the resistance spectrum. Interestingly, mutations causing clarithromycin resistance are different in Asia compared to those observed in Europe and North America [55]. In Western countries, approximately 90% of clarithromycin‐resistant strains have the A2142G or A2142C mutation [56], while this mutation accounts for only 23% of resistant strains in Asia. Additionally, other mutations, such as T2183C and A2223G, are more common in Asian populations [57]. In a study conducted by Kulmagambetova et al., 87.5% of samples taken from Kazakh residents had mutations in the 23S rRNA gene, as detected by PCR and sequencing. Of these, 56.25% had the T2182C mutation, and 12.5% had either the A2142G or A2143G mutations [58], which is similar to findings from foreign studies.

The isolation and cultivation of *H. pylori* from gastric biopsy samples and antimicrobial susceptibility testing can be challenging. The results of the study were influenced by several factors, including the quality of clinical samples, contamination, transportation, and personnel skills. Microbiological cultivation of *H. pylori* is a less sensitive, but highly specific, method with a specificity rate of 100%. This technique also allows for the determination of antimicrobial susceptibility [59]. Factors that can influence culture results include a low bacterial load, the use of PPIs and antibiotics, alcohol, and bleeding. Additionally, there may be false‐negative results due to incorrect or inadequate specimen collection, transportation delays, exposure to air, or lack of experience on the part of the microbiologist. For the first time in Kazakhstan, a cultural study of *H. pylori* was carried out in 2011. This study examined 16 strains of *H. pylori* isolated from biopsy specimens of the gastric mucosa of symptomatic patients. *H. pylori* was cultivated on Columbia agar under microaerophilic conditions, and DNA was subsequently extracted from the isolated strains. The practical significance of this method lies in the possibility of determining the spectrum of antibiotic resistance of *H. pylori* . In this study, 87.5% of the strains showed resistance to clarithromycin, 31.25% to metronidazole, and 18.75% to amoxicillin [39]. This study was a pilot study with a limited number of samples. Due to the burden of *H. pylori* infection in Kazakhstan, the Science Committee of the Ministry of Education and Science has allocated grant funding for research on *H. pylori* . As part of this research, in the near future, data will be collected in central Kazakhstan on culturing *H. pylori* and determining its sensitivity to antibacterial agents.

The rapid urease test (RUT) is one of the most common methods for detecting *H. pylori* infection worldwide, but it is not the gold standard for diagnosis [59]. A disadvantage of RUT is the potential for false‐negative and false‐positive results. The causes of the false‐negative test results were similar to those of the UBT. False‐positive results can be caused by the presence of other bacteria that produce urease, such as *Proteus mirabilis* [1]. In Kazakhstan, despite a lack of studies using RUT, this method of bacterial detection is often used in regular gastroenterology services, as endoscopy of the stomach is covered by the State Medical Care Fund and patients can undergo RUT tests free of charge during endoscopic examinations. A study by Malfertheiner et al. found that the specificity of RUT was 95%–100%, indicating that false positive results are rare [1]. The cost of the rapid urease test is included in the price of the endoscopic examination of the stomach, which is part of the overall fee for the endoscopy procedure and amounts to $14.53.

The stool antigen test (SAT) method has several advantages: it is a noninvasive procedure, easily transportable material, does not require expensive special equipment, and has a low price compared to other noninvasive methods. It also does not require fasting or the withdrawal of PPIs when using new monoclonal antibodies. Despite these advantages, some disadvantages should be considered. Antigen excretion can vary over time and antigens can be destroyed during intestine transit. Additionally, the use of a mucolytic agent such as N‐acetylcysteine can reduce the accuracy of the diagnosis [60]. Furthermore, low bacterial colonization in the stomach can lead to a decreased concentration of *H. pylori* antigens in samples [61]. Although SAT has not been widely used in research studies in Kazakhstan, it is available for outpatient. Therefore, we anticipate the emergence of publications on the study of these methods in the Kazakh population. It is a less expensive option than UBT, with the cost an average of $10. Despite the frequent use of SAT as a diagnostic tool for *H. pylori* infection, the cost of this procedure is not covered by insurance and is not included within the standard scope of medical services. Therefore, self‐paying patients may not be able to access this test. Additionally, the practical benefit of this method is its ability to diagnose *H. pylori* infection and assess its effectiveness of treatment. In light of the information provided above, UBT and SAT have been increasingly utilized in clinical practice. Nevertheless, the cost of these procedures, which are not covered by the fee schedule for treated patients, is a reason for their absence from the guaranteed volume of medical care; therefore, they are not always available to self‐paying patients.

International guidelines for the management of *H. pylori* infection require two positive tests to establish a diagnosis. In practice, this requirement is fulfilled by the histological confirmation of *H. pylori* infection, combined with chronic active gastritis. The latter provides additional evidence of bacterial infection of the gastric mucosa [60]. According to international consensus reports, patients aged 50 years or older with a primary diagnosis of *H. pylori* infection are recommended to undergo endoscopy and histological examination for accurate classification of gastritis. All guidelines support the use of the operating link for gastritis assessment (OLGA) or operating link for gastric intestinal metaplasia (OLGIM) risk stratification schemes, which allow the assessment of the risk of gastric cancer based on the severity, extent, and location of gastric atrophy [62]. This method was not used in any of the studies listed in Kazakhstan. However, this study is currently being conducted at both the republican and regional centers of Kazakhstan. On average, histological examination for *H. pylori* costs approximately $38.41.

## 4 Conclusions

In Kazakhstan, invasive tests for the detection of *H. pylori* infection are not widely used. Preference is given to noninvasive tests, such as UBT and SAT. There are challenges in determining *H. pylori* 's susceptibility to antibiotics and its genotyping. The most accessible method for diagnosing *H. pylori* infection in Kazakhstan is ELISA.

## Conflicts of Interest

The authors declare no conflicts of interest.
